# Terminal Dopamine Release Kinetics in the Accumbens Core and Shell Are Distinctly Altered after Withdrawal from Cocaine Self-Administration

**DOI:** 10.1523/ENEURO.0274-16.2016

**Published:** 2016-10-06

**Authors:** Michael P. Saddoris

**Affiliations:** Department of Psychology & Neuroscience, University of Colorado Boulder, Boulder, CO 80309

**Keywords:** dopamine transporter, drug addiction, Michaelis–Menten, plasticity, ventral striatum, voltammetry

## Abstract

Repeated self-administration of cocaine is associated with impairments in motivated behaviors as well as alterations in both dopamine (DA) release and neural signaling within the nucleus accumbens (NAc). These impairments are present even after several weeks of abstinence from drug taking, suggesting that the self-administration experience induces long-lasting neuroplastic alterations in the mesolimbic DA circuit. To understand these changes at the terminal level, rats were allowed to self-administer either cocaine intravenously (∼1 mg/kg per infusion) or water to a receptacle (control) in 2-h sessions over 14 days, followed by 30 days of enforced abstinence. Fast-scan cyclic voltammetry was used to record real-time DA release in either NAc core or shell after electrical stimulations of the ventral tegmental area (VTA) in freely-moving animals. In controls, the kinetics of DA release in the core and shell strikingly differed, with shell displaying slower release and reuptake rates than core. However, cocaine experience differentially altered these signaling patterns by NAc subregion. In the shell, cocaine rats showed less sensitivity to the dynamic range of applied stimulations than controls. In the core, by contrast, cocaine rats displayed robustly reduced peak DA release given the same stimulation, while also showing slower release and reuptake kinetics. The differential effects of cocaine self-administration on terminal function between core and shell is consistent with a region-specific functional reorganization of the mesolimbic DA system after repeated exposure and may provide an anatomical substrate for altered cognitive function after chronic drug-taking and addiction.

## Significance Statement

Chronic drug use alters neural signaling (particularly dopamine), even after extended periods of drug abstinence. Evidence suggests that dopamine terminals may be persistently altered in cocaine-experienced animals (i.e., influencing the rates and amount of dopamine release and reuptake), but it is not known whether this is a general property of the dopamine system or whether changes are unique within different terminal regions. Voltammetric recordings in the nucleus accumbens core and shell in cocaine-experienced rats revealed region-specific differences in release/reuptake kinetics relative to controls. Strikingly, whereas drug-naive subjects showed consistent differences in dopamine kinetics between core and shell, cocaine remodeled the entire accumbens to become more “shell-like.” Understanding this remodeling will be critical for developing treatments to prevent drug relapse.

## Introduction

Phasic dopamine (DA) signaling in the nucleus accumbens (NAc) is implicated in learning, motivation, reward encoding, and drug taking (Schultz et al., 1997; Berridge and Robinson, 1998; Berridge, 2012; Berridge and Kringelbach, 2015; Saddoris et al., 2015a). Evidence suggests that DA signaling acts to modulate activity of NAc neurons by permitting plasticity for task-relevant stimuli. For example, in NAc, phasic patterns of neural activity arise only in regions where phasic DA signals are also present (Cheer et al., 2005, 2007; Owesson-White et al., 2009), whereas blockade of the DA signal via AP-5 in the ventral tegmental area (VTA) abolishes phasic excitatory encoding in NAc neurons (Cacciapaglia et al., 2011).

Growing evidence suggests that cocaine use differentially acts on the DA system in the NAc. For example, rats willingly self-administer cocaine into the NAc shell but not core (Rodd-Henricks et al., 2002; Ikemoto, 2003). Behaviorally, although normal DA signaling encodes information about task-relevant stimuli, animals with a history of cocaine self-administration display abnormal phasic DA release patterns, even after several weeks of drug abstinence, that strikingly differ between core and shell (Saddoris et al., 2016b). Thus, because both acute and chronic actions of repeated cocaine experience differentially alter DA release dynamics and related associative neural encoding within neuroanatomically distinct terminal regions (Saddoris and Carelli, 2014), it is essential to understand how drug experience may uniquely alter DA signaling in core and shell.

However, it can be difficult to determine whether altered phasic DA signaling is due to changes in (1) the ability for DA neurons to appropriately encode task-relevant information (i.e., disruptions of limbic inputs to the VTA); (2) the ability for DA neurons to appropriately release DA (i.e., disruptions of output of VTA neuron terminals within the NAc); or (3) some combination of the two. We and others (Willuhn et al., 2014) have shown that cocaine alters phasic DA signaling during behavior, but other studies have indicated that DA terminal function is significantly altered as well (Jones et al., 1996; Mateo et al., 2005; Yorgason et al., 2011; Calipari et al., 2014; Siciliano et al., 2015). In those studies, however, DA kinetics were often examined in *ex vivo* brain slice preparations (e.g., Ferris et al., 2013), which may differ from how these systems may operate in awake and behaving animals. Further, although some of these experiments have examined how cocaine exerts long-term effects after prolonged drug abstinence (Cameron et al., 2016; Siciliano et al., 2016), none have investigated whether the extended withdrawal from drug taking differentially affects DA signaling in core and shell.

To isolate the question of terminal function, I implanted electrical stimulation probes into the VTA of freely moving 30-d abstinent rats with a history of cocaine self-administration or drug-naive controls and voltammetrically assessed the real-time kinetics of the phasic DA signal in the NAc after variations of applied stimulation frequencies and durations. Critically, voltammetry recordings were taken from both core and shell, allowing for isolation of the effects of cocaine experience on terminal function in these regions. Whereas DA release kinetics were changed in both core and shell after cocaine self-administration experience, core kinetics were altered in a manner that resembled the shell in drug-naive rats across several metrics. Thus, cocaine experience appears to differentially augment DA terminal function between core and shell that persists long after the cessation of drug taking.

## Methods

### Subjects

Male Sprague-Dawley rats (*n* = 31) were used and lightly food-deprived to ∼90% of their free-feeding weight at the time of recording (Charles River Laboratories, Wilmington, MA). During all phases of the experiment, single-housed rats were allowed *ad libitum* access to water in their home cages and maintained on a 12:12 light:dark schedule. Stimulations were obtained from subjects trained in appetitive conditioning experiments. Recordings during the associated behavioral experiments and descriptions of those tests appear elsewhere (Sugam et al., 2012; Saddoris et al., 2015a, 2015b). Experiments were performed in accordance with University of North Carolina (UNC) Chapel Hill Institutional Animal Care and Use Committee protocols (12-236, 11-057, and 09-240).

### Behavior

#### Self-administration

**Figure 1.**
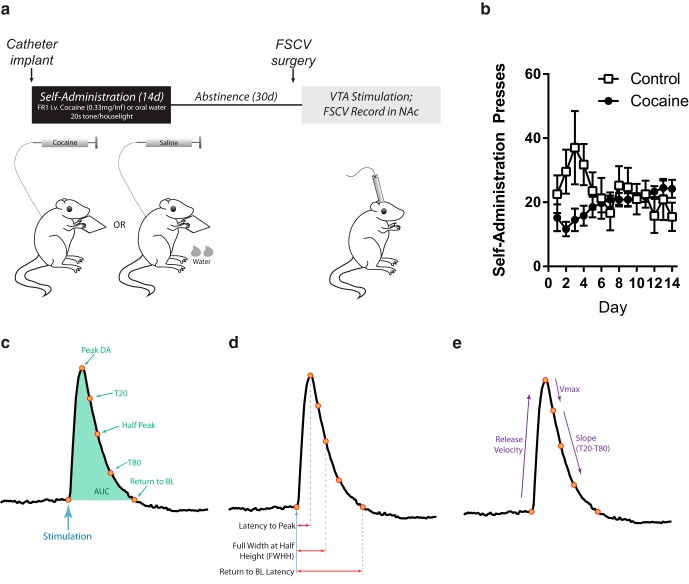
Schematic of experimental design, reinforced presses across the 14 d of self-administration training, and schematic of different metrics of DA release kinetics. ***A***, Points in the release kinetics in relation to the peak DA release (i.e., point of greatest [DA] after stimulation). Half peak is the point at exactly half of peak concentration, Return to baseline (BL) is the point at which the [DA] was within a 95% confidence interval of the baseline, and T20 and T80 reflect 20% and 80% decrease in [DA] from peak, respectively. AUC was estimated by summing the [DA] in each 100-ms bin between stimulation and return to BL. ***B***, Latency measures derived from the points of release and reuptake from ***A***. Latency to peak, FWHH (i.e., latency from stimulation to half peak), and return to BL latency are relative to stimulation, whereas T20 and T80 latencies are relative to peak. ***C–E***, Rates of change relative to points during release. Release velocity is the rate of increase in [DA] from stimulation to peak, V_max_ is the rate of uptake between the peak and T20, and slope is the rate of uptake between T20 and T80.

Detailed descriptions of this task appear elsewhere (Saddoris and Carelli, 2014; Saddoris et al., 2016b). Briefly, at least 1 month before testing, a subset of rats (*n* = 22) were implanted with intrajugular catheters. After recovery, rats were randomly assigned to either the intravenous cocaine self-administration group (cocaine; *n* = 10) or water self-administration group (control; *n* = 12). Cocaine was provided by the NIDA Drug Supply Program (National Institute on Drug Abuse, NIH, Bethesda, MD). All self-administration sessions were performed in a standard rat chamber (Context A: 25 × 25 × 30 cm, stainless steel rod floor; Med Associates, St Albans, VT). For the cocaine subjects (Fig. 1*A*), pressing on a lever below an illuminated cue light resulted in an infusion of intravenous cocaine (0.33 mg/infusion; ∼1 mg/kg) coupled with a 20-s presentation of a house light and intermittent tone, extinguishing of the cue light, and retraction of the lever. For the controls (Fig. 1*A*), pressing on the lever under the illuminated cue light resulted in the same stimuli (house light/tone, lever retraction, and cue light extinguishing), but the reinforcer was water (250 μl) delivered to a centrally located food cup. Controls also received yoked saline infusions based on the self-administration schedule of a cocaine rat in an adjacent box. Both groups were allowed to press for 2 h per session for 14 sessions. After this, all rats entered a period of enforced abstinence for 30 d by remaining in their home cages in the colony room with ad libitum access to food and water.

#### Previous training

The group of drug-naive rats that did not receive jugular catheters (*n* = 9) had been previously trained to perform an instrumental discrimination; the results and descriptions of those experiments appear elsewhere (Sugam et al., 2012; Saddoris et al., 2015b). Briefly, rats in this task learned that pressing on one lever resulted in one type of reward option (one pellet), while pressing on the other lever resulted in a different reward option (a larger food reward with either a delay or decreased probability of delivery). There was no effect of previous experience on any measure of DA (water control vs. drug-naive control, *F*_(1,281)_ = 0.062, *p =* 0.80), and as such both groups were collapsed into a larger control group for all subsequent analyses (12 controls plus nine drug-naive controls = 21 controls). Note that for a subset of subjects (*n* = 8 control; *n* = 3 cocaine), two recordings were taken in the same animal. Critically, the second recording was at least 300 μm ventral to the first, ensuring that the recording was taken from new tissue.

#### Fast-scan cyclic voltammetric recordings

Fast-scan cyclic voltammetric (FSCV) recordings were performed in awake and behaving rats identical to those described previously (Sugam et al., 2012). Briefly, a carbon fiber electrode was acutely lowered into the NAc core or shell using a custom manipulator, then locked in place. An Ag/AgCl-plated reference wire was inserted at the time of recording in the contralateral hemisphere. Both the electrode and reference were connected to an amplifying headstage (UNC Chemistry, Chapel Hill, NC). Changes in current were detected by applying ramping voltage (from –0.4V to +1.3V and back to –0.4V over 10 ms); this change was detected by software, and chemometrics were used to convert current into DA release concentrations at the recording site using HDCV Analysis software (UNC, Chapel Hill, NC). To ensure reliable comparisons between groups on measures of peak and area under the curve, the average baseline concentration before the event of interest (pellet delivery, stimulation) was subtracted from the concentration in each bin during the effect period. This ensured that the average baseline for each trial would be set to 0 nm, thereby isolating the absolute change in [DA] as a result of the event. Likewise, this set the cumulative DA release during the baseline to 0 nm, again effectively isolating the absolute change in cumulative [DA] release.

DA release was elicited by electrical stimulation of VTA afferents via the bipolar stimulating probe. These were generated for each subject in the course of developing a training set specific for each electrode and at each recording location (Rodeberg et al., 2015). Bipolar stimulations consisted of a series of pulses (2 ms positive, 2 ms negative for a total pulsewidth of 4 ms per pulse), which varied in both frequency and number. The range of frequencies applied spanned 12–60 Hz, whereas the number ranged from 1 to 24 pulses. To simplify this range to a single dimension, a stimulation index was used, which is the product of frequency × pulse number (e.g., a stimulation delivered at 20 Hz for 10 pulses would result in a stimulation index of 20 × 10 = 200). Each subject received multiple stimulations that sampled throughout the stimulation index range (20–1440) for an average of 16 ± 6 stimulations per subject.

### Determinants of DA release and reuptake kinetics

To understand the kinetics of DA release and reuptake, several metrics were adopted from those described in detail in Yorgason et al. (2011). These factors are shown in Fig. 1*C*–*E*. First, several points were established in the DA release curve (Fig. 1*C*). For each trial, electrical stimulations occurred after a 5-s baseline period, followed by 10 s of a poststimulation period. Peak DA was the greatest concentration of DA release within 3 s after stimulation. Other points examined reuptake relative to the peak level. Half-peak was the point in the reuptake that was half of peak concentration, whereas T20 and T80 were periods that indicated 20% and 80% decay from peak, respectively. Finally, a 95% confidence interval around the 5-s baseline period was established for each trial, and then the first point during reuptake was computed where the [DA] returned this confidence interval following peak.

Based on these points, the latency at which the DA signal reached these points was computed (Fig. 1*D*). Latency to peak was the time elapsed between stimulation and peak. Other factors measured relative to stimulation onset included the latency to half-peak (i.e., full width at half-height; FWHH), and the latency to the return to baseline (within 95% confidence interval of baseline). Finally, the rates of change in [DA] between points were computed. These included release velocity (i.e., the rate of increase in [DA] between stimulation and peak), slope (here, the average rate of uptake between T20 and T80), and V_max_ (here, the maximum rate of uptake as estimated by the rate of change between peak and T20). Note that V_max_ in this case is not a true measure of maximum reuptake, which can only truly be computed with Michaelis–Menten equations when the DA transporter (DAT) is saturated. Although this may be the case at the very high stimulation levels, it is not certain for any of the recordings in awake and behaving rats. Further, the maximum rate of postpeak reuptake in all of the samples is of interest, not just the very large (and physiologically unrealistic) stimulations. Thus, the measure of V_max_ is an estimate of this function rather than a true V_max_, but it captures an important aspect of reuptake kinetics. In contrast, the other measures presented here are not dependent on DAT saturation for accurate computation (Yorgason et al., 2011) and are presented without correction.

All factors were determined using equations based on the above criteria and were thus unbiased by group or region.

### Statistical analysis

The shape of the stimulated DA traces are heavily influenced by a number of factors which tend to scale with the magnitude of the peak DA level (e.g., the latency from stimulation to a return to a postpeak baseline will positively correlate with the height of the peak [DA]). As such, to determine with more certainty how these factors compare, the observations were equated by two factors: peak and stimulation intensity. For peak magnitude alignments, blocks were aligned by peak responses, and were defined as low peak (<0.1 μm DA), medium-low peak (0.1–0.2 μm DA), medium-high peak (0.2–0.4 μm DA), and high peak (0.4–0.8 μm DA). Within these blocks, then, all observations were matched for peak, thus allowing for more controlled comparison of other factors (e.g., FWHH, latency to peak). For the stimulation intensity, stimulation index (frequency × pulse number) was used. Blocks were low frequency (stimulation index 40–100), medium-low frequency (stimulation index 100–300), medium-high (stimulation index 300–600), and high frequency (stimulation index >600). In general, blocks were chosen based on the relative frequency of observations between groups to ensure relatively equivalent numbers of stimulations between groups.

Each analysis used individual stimulations based on the block criteria, region (core or shell), and drug background (cocaine or control). Each kinetic factor was thus subject to a multifactorial ANOVA that used either drug background or region as one factor and block as the other factor. Note that given the variability in the number of observations for any given bin within a group and/or block, unequal *n* was corrected for by using a weighted mean (Type III) sum of squares in the analyses. For significant main effects or interactions of either drug background or region, pairwise comparisons between the groups at each level of the block with *t* tests were used as a post hoc test. *T* tests were chosen as a post hoc test because experiment-wise post hoc tests (e.g., Tukey honestly significant difference) use a single determinant to estimate significance based on expected pairwise differences. As such, these tests will underestimate reliable differences at low stimulations and peaks while overestimating differences at high stimulations and peaks. Therefore, *t* tests at each level were independent of experiment-wise variance and isolated the specific effects at a given level. Critically, a Bonferroni correction was used for these *t* tests to control for multiple comparison error. In addition, significant main effects of block and interactions of block by region/drug orthogonal linear contrasts were used to determine whether the rates of change in the kinetic factor differed by region or drug background. Statistics for ANOVAs and pairwise comparisons were done using Statistica (v. 12; StatSoft, Tulsa, OK) and **χ** ^2^ analysis was done using QuickCalcs (GraphPad, La Jolla, CA). Graphs were generated using GraphPad Prism 6.

## Results

Data were obtained from recordings in 31 rats, which included nine rats that were naive to self-administration, 12 that were water self-administration controls (thus a total of 21 controls), and 10 with a history of cocaine self-administration.

For rats with a history of self-administration, rates of self-administration pressing were similar between cocaine subjects and controls, particularly by the end of training when pressing rates were stable (Fig. *1*B). Rates of self-administration of cocaine were similar to those from previous reports that were sufficient to augment both DA release and neural signaling in the NAc (Saddoris and Carelli, 2014; Saddoris et al., 2016b). There was a significant interaction of drug (cocaine versus water) × day, *F*_(13, 143)_ = 2.76, though pairwise post hoc comparisons between groups failed to find any significant differences in press rate on any day of conditioning (Tukey: all *p* > 0.65). Critically, there were no effects of region (rats that were destined to have recordings in the core or shell) or interactions of region with any other factor (all *p* > 0.65), indicating that all subjects had equivalent training and experience with self-administration before recordings.

**Figure 2.**
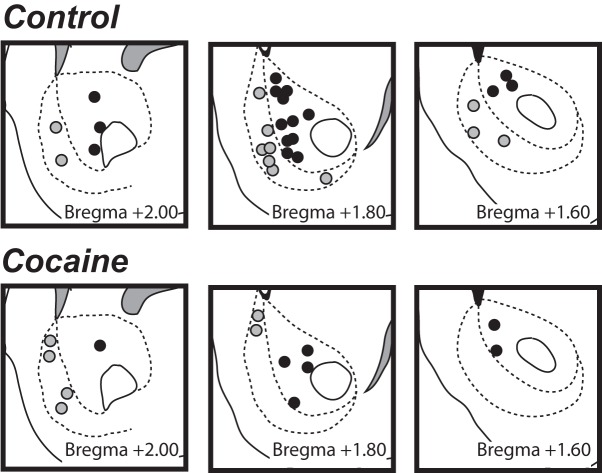
Placement of electrodes during recording in controls (top) or cocaine (bottom) rats. Black circles, core; gray circles, shell.

Histological placements of carbon fiber electrode tips in the NAc (Fig. 2) indicated recordings from 24 locations in the core (*n* = 17 in controls, *n* = 7 in cocaine) and 18 locations in the shell (*n* = 12 in controls, *n* = 6 in cocaine). From these, I obtained 218 stimulation trials from the core of controls and 102 stimulations of cocaine rats, and 112 stimulation trials from the shell of controls and 63 of cocaine rats.

**Figure 3.**
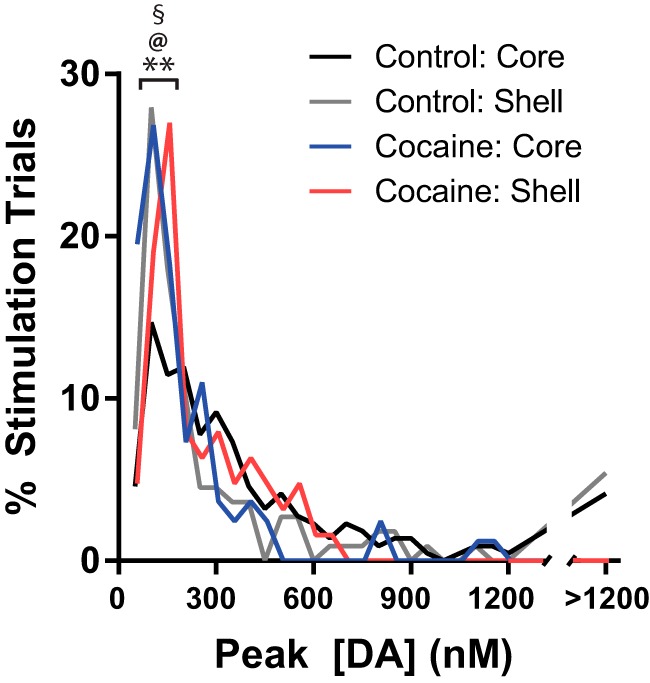
Distribution of peak [DA] amplitude from stimulation trials in the NAc core (control, black; cocaine, blue) and NAc shell (control, gray; cocaine, red). Peak [DA] responses for each stimulation were binned by 50-ms epochs from 0 to 1200 nm, while all stimulations that were greater than 1200 nm represented the final bin. Proportion reflected the number of stimulations in that bin as a proportion of all stimulations from that group. **Control core vs. control shell; ^§^control core vs. cocaine core; ^@^control shell vs. cocaine shell; *p* < 0.001 for relevant χ^2^.

Stimulations were quantified based on the distribution of peak DA responses from each group. Peak DA stimulations were first binned in increments of 50 nm from 0 to 1200 nm, with a final aggregate bin comprised of all stimulations with peak DA greater than 1200 nm (Fig. 3). In controls, the distribution of peak DA in the shell after stimulations was skewed toward lower peaks (median, 125.7 nm) compared to the core, which were more evenly spread across the distribution space (median, 248.2 nm). Indeed, the number of stimulations with a peak response lower than 150 nm was reliably greater in shell than in core relative to the residual of the populations (**χ**^2^ = 15.91, *p* < 0.0001). In contrast, the distribution of peak DA in the core and shell after stimulation in cocaine rats showed a different pattern. In cocaine subjects, the distribution of peak DA was similar between core and shell (median cocaine core, 108.5 nm; median cocaine shell, 145.6 nm), whereas both groups displayed distributions that closely resembled that seen in the shell of controls (median, 125.7 nm). Indeed, both cocaine groups showed significantly greater numbers of peaks less than 150 nM than the core controls (core control vs. core cocaine, **χ**^2^ = 27.14, *p* < 0.0001; core control vs. shell cocaine, **χ**^2^ = 7.76, *p* = 0.0053), and neither cocaine group differed between shell control in proportion of peak stimulations less than 150 nM (shell control vs. shell cocaine, **χ**^2^ = 0.66, *p* = 0.80; shell control vs. core cocaine, **χ**^2^ = 1.76, *p* = 0.18).

Observations were then binned into larger blocks by peak DA (0–0.1 μm [low], 0.1–0.2 μm [medium-low], 0.2–0.4 μm [medium-high], 0.4–0.8 μm [high], and >0.8 μm [very high]) to assess whether there were differences at the higher peaks that were not immediately discernable with 50-nM bins (data not shown). Here, the previous observation was replicated that there were significantly more low peak stimulations in the shell than core in controls (low block, **χ**^2^ = 10.18, *p* = 0.0014), but core stimulations produced a greater number of higher peaks than shell in the medium-high block (**χ**^2^ = 5.33, *p* = 0.021) and a nearly significant trend in the high peak block (**χ**^2^ = 3.73, *p* = 0.053). However, cocaine experience significantly shifted this distribution downward in the core. As a result, there were more stimulations that elicited low peaks in the core of cocaine animals than controls (low peak block, **χ**^2^ = 19.22, *p* < 0.0001), and fewer higher-magnitude peaks (high peak block, **χ**^2^ = 8.01, *p* = 0.002; very high block, **χ**^2^ = 13.98, *p* = 0.0001). In contrast, the distribution of peak responses in the shell was less affected by cocaine. There were no differences between control and cocaine groups in any bin less than 0.8 μm (all *p* > 0.13), though cocaine appeared to have eliminated the very high peaks seen in controls (**χ**^2^ = 4.47, *p* = 0.03). Interestingly, there were no differences in distributions between shell controls and core cocaine in any block (all *p* > 0.11). Indeed, the only difference between the shell control and combined cocaine groups (cocaine core plus cocaine shell) was at the very high block (**χ**^2^ = 6.75, *p* = 0.01; all others, *p* > 0.25), whereas there were robust differences between core control and the combined cocaine groups (low, **χ**^2^ = 11.65, *p* = 0.0006; high, **χ**^2^ = 4.98, *p* = 0.03; very high, **χ**^2^ = 8.65, *p* = 0.003). Thus, the distribution of peak responses in cocaine-experienced animals was much more consistently similar to that normally found in the shell, but distinctly unlike that typically found in the core.

### Differential core and shell release kinetics in controls

**Figure 4.**
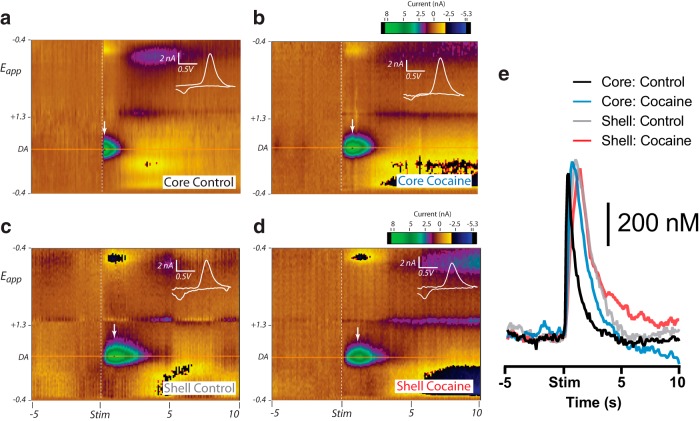
Representative color plots of stimulated DA release in NAc core (***A*** and ***B***) and NAc shell (***C*** and ***D***). ***E***, Overlapped traces of DA elicited by electrical stimulation in core and shell of controls and cocaine-experienced subjects from the representative color plots in ***A–D***.

It was next important to understand whether release and reuptake kinetics differed by region and cocaine experience. However, because many factors in these measures can be intrinsically correlated (e.g., larger peaks will also typically show a slower return to baseline), it was important to control for at least one factor when making comparisons between observations. Thus, data were compared using two organizing principles. First, data were grouped based on peak DA (as above) regardless of stimulation intensity. However, because the extremely few observations in the very high block, analysis was performed within and across four blocks (low, medium-low, medium-high, and high peak) and between regions (core, shell) and drug history (control, cocaine). Then, these same data were grouped based on stimulation intensity (regardless of peak) based on the stimulation index (frequency × number of pulses), also grouped by a four-block design (low, medium-low, medium-high, and high stimulation). Representative color plots from the core and shell in control and cocaine groups are shown in Fig. 4*A*–*D*.

Peak-aligned stimulated DA events revealed multiple factors that differed between core and shell. Despite similar peaks, multiple measures of response kinetics in the shell in controls were reliably slower than in the core. However, after cocaine experience, both core and shell kinetics more obviously resembled normal shell responses (Fig. 4*E*). This was formalized by running a three-way ANOVA that used group (core control, core cocaine, shell control, shell cocaine) and blocks of peak DA height (low, medium-low, medium-high, and high) as factors across a variety of kinetics measures. In general, on the majority of these measures, peak-aligned DA responses supported the hypothesis that cocaine experience shifted core DA release kinetics into a more shell-like pattern. For pairwise *t* test comparisons between groups, see Tables 1 and 2 for Bonferroni-corrected *p*-values.

**Table 1.** Peak-aligned pairwise comparisons (individual drug groups)

*p*-values (*t* test)	**Core (control) vs. shell (control)**				**Core (control) vs. core (cocaine)**				**Shell (control) vs. shell (cocaine)**			
Peak [DA], µm	**0. 1**	**0.2**	**0.4**	**0.8**	**0.1**	**0.2**	**0.4**	**0.8**	**0.1**	**0.2**	**0.4**	**0.8**
Peak	0.87	*0.02*	0.97	0.32	*0.03*	0.001*	0.07	0.66	0.81	0.42	0.94	*0.03*
Frequency	0.007*	0.001*	0.54	0.91	*0.04*	0.28	0.10	0.85	*0.03*	0.20	0.43	0.34
AUC	*0.02*	*0.05*	0.001*	0.008*	0.06	0.12	0.11	0.09	0.10	0.52	0.88	0.24
Rise velocity	<0.0001*	<0.0001*	<0.0001*	0.004*	0.002*	<0.0001*	0.008*	0.07	0.0004*	0.06	0.06	0.07
Latency peak	<0.0001*	<0.0001*	<0.0001*	<0.0001*	0.27	<0.0001*	0.12	0.003*	<0.0001*	*0.02*	0.22	0.53
V_max_	<0.0001*	<0.0001*	<0.0001*	0.01	0.0008*	<0.0001*	*0.02*	0.08	0.63	0.52	0.53	0.36
FWHH	<0.0001*	<0.0001*	<0.0001*	<0.0001*	0.28	<0.0001*	0.15	0.003*	<0.0001*	0.14	0.67	0.93
Slope (T20–T80)	<0.0001*	<0.0001*	0.52	0.80	0.84	0.05	0.98	0.98	0.41	0.37	0.21	0.009*
Baseline return	0.0002*	0.002*	0.60	0.63	0.84	0.83	0.56	0.34	0.87	0.23	0.34	0.35
T20 latency	<0.0001*	<0.0001*	<0.0001*	<0.0001*	0.16	<0.0001*	0.12	<0.0001*	<0.0001*	0.07	0.69	0.89
T80 latency	<0.0001*	<0.0001*	*0.02*	0.85	0.91	0.10	0.80	0.40	0.0006*	0.87	0.41	0.08

Significance (*p*-value) of pairwise *t* tests at each peak bin (low [<0.1 μm DA], medium-low [0.1–0.2 μm DA], medium-high [0.2–0.4 μm DA], and high [0.4–0.8 μm DA]) between core control and shell control (left), core control and core cocaine (middle), and shell control and shell cocaine (right). Bold italics: *p < 0.01 (significant after Bonferroni correction); italics only: *p* < 0.05 (not significant after Bonferroni correction).

**Table 2.** Peak-aligned pairwise comparisons (collapsed drug groups)

*p*-values (*t* test)	**Core (control) vs. Shell (control)**				**Core (control) vs. both cocaines**				**Shell (control) vs. both cocaines**			
Peak [DA], µm	**0. 1**	**0.2**	**0.4**	**0.8**	**0.1**	**0.2**	**0.4**	**0.8**	**0.1**	**0.2**	**0.4**	**0.8**
Peak	0.87	0.23	0.97	0.32	0.11	<0.0001*	0.28	0.26	0.86	0.24	0.49	0.13
Frequency	0.007*	0.002*	0.54	0.91	0.001*	0.12	0.18	0.34	0.66	0.10	0.13	0.53
AUC	*0.02*	*0.03*	0.001*	0.008*	0.98	0.72	0.20	0.07	0.05	*0.04*	0.07	0.33
Rise velocity	<0.0001*	<0.0001*	<0.0001*	0.004*	<0.0001*	<0.0001*	<0.0001*	<0.0001*	0.07	0.08	0.82	0.17
Latency peak	<0.0001*	<0.0001*	<0.0001*	<0.0001*	0.0004*	<0.0001*	<0.0001*	<0.0001*	0.67	*0.70*	0.37	0.96
V_max_	<0.0001*	<0.0001*	<0.0001*	0.01	<0.0001*	<0.0001*	<0.0001*	0.0008*	*0.05*	0.20	0.48	0.35
FWHH	<0.0001*	<0.0001*	<0.0001*	<0.0001*	0.0004*	<0.0001*	<0.0001*	<0.0001*	0.32	0.47	0.12	0.64
Slope (T20–T80)	<0.0001*	<0.0001*	0.52	0.80	0.16	0.001*	0.10	0.19	0.003*	0.08	0.27	*0.01*
Baseline return	0.0002*	0.002*	0.60	0.63	0.16	0.30	0.49	0.87	0.007*	0.05	0.99	0.70
T20 latency	<0.0001*	<0.0001*	<0.0001*	<0.0001*	0.0002*	<0.0001*	<0.0001*	<0.0001*	0.62	0.50	0.07	0.71
T80 latency	<0.0001*	0.0006*	*0.02*	0.85	0.0008*	0.0001*	0.007*	0.001*	0.06	0.25	0.27	0.16

Significance (*p*-value) of pairwise *t* tests at each peak bin (low [<0.1 μm DA], medium-low [0.1–0.2 μm DA], medium-high [0.2–0.4 μm DA], and high [0.4–0.8 μm DA]) between core control and shell control (left; repeated from Table 1), core control and average of both cocaine groups (core and shell; middle), and shell control average of both cocaine groups (core and shell; right). Bold italics: *p < 0.01 (significant after Bonferroni correction); italics only: *p* < 0.05 (not significant after Bonferroni correction).

**Figure 5.**
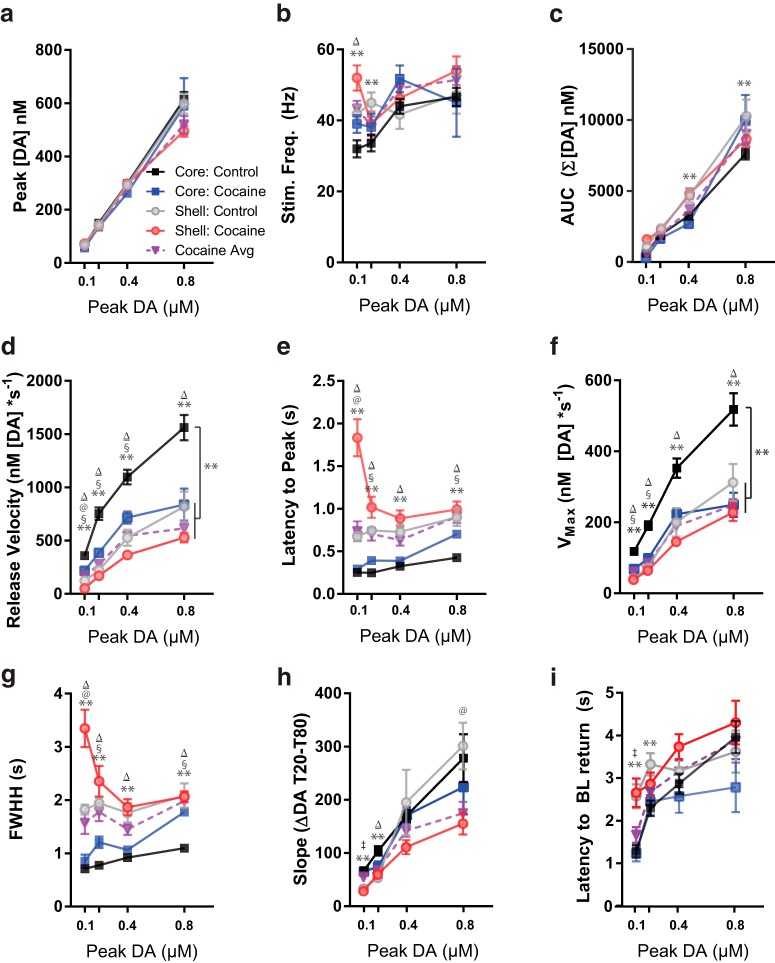
Kinetic factors of DA release aligned by peak [DA] in control core (black squares), cocaine core (blue squares), control shell (gray circles), and cocaine shell (red circles) recordings. **Control core vs. control shell; ^Δ^control core vs. both cocaines; ^§^control core vs. cocaine core; ^@^control shell vs. cocaine shell; ^‡^control Shell vs. both cocaines; *p* < 0.01 (Bonferroni-corrected α for multiple comparisons).

First, it was important to show that aligning by peak resulted in similar groups of data within blocks across treatment groups (Fig. 5*A*). This was largely true, although there was a modest interaction between group × block [*F*_(9,425)_ = 2.25, *p* = 0.02]. However, no post hoc pairwise comparisons reached significance at any block between groups or drug background, indicating that peak DA was consistent across all groups and blocks, and therefore allowing for direct comparisons of kinetics of stimulations with matched peaks.

Several kinetic factors were then explored. First, applied stimulation frequency (Fig. 5*B*) indicated a modest interaction of group × block, *F*_(9,425)_ = 1.94, *p* = 0.045, which was due largely to core controls showing lower applied frequencies in the low peak block than both shell controls (*p* = 0.007) and both cocaine groups (*p* = 0.001). In contrast, the shell controls were not different from both cocaine groups in this block (*p* = 0.66). Further, planned linear contrasts indicated that core controls showed a linear increase in peak as a function of frequency [*F*_(1,425)_ = 26.8, *p* < 0.0001], while no other group showed any such linear response (all *p* > 0.17). Indeed, the orthogonal linear contrast between Core control versus all other groups was significant [*F*_(1,425)_ = 12.4, *p* = 0.0005], while the contrast between shell control and both cocaine groups was not [*F*_(1,425)_ = 0.03, *p* = 0.85]. Thus, although core controls showed linear increases in peak with increases in applied stimulation frequency, all other groups were less dynamically related to this parameter.

Next, the total DA release between stimulation and the return to baseline was measured (area under the curve [AUC]; Fig. 5*C*). Despite similar peaks, there was a significant main effect of group, *F*_(3,425)_ = 15.71, *p* < 0.00001, which indicated a significant pairwise difference between core controls and core cocaine (*p* = 0.00001), but no difference between shell controls and shell cocaine (*p* > 0.10). There was a further group × block interaction [*F*_(9,425)_ = 3.70, *p* = 0.0002]. Specifically, while all groups showed significant linear increases in AUC across blocks (all *p* < 0.00001), core controls increased at a slower rate across blocks than core cocaine [*F*_(1,425)_ = 8.50, *p* = 0.004] and shell controls [*F*_(1,425)_ = 18.29, *p* = 0.00002], and there was no difference in the linear change across blocks between the shell controls and core cocaine [*F*_(1,425)_ = 0.04, *p* = 0.84]. Consistent with previous findings, core controls showed consistently smaller AUC compared with shell controls, particularly in the large peak block (*p* = 0.008), whereas shell controls did not show a difference in AUC compared to either cocaine group (*p* = 0.23 shell cocaine; *p* = 0.90, core cocaine) in this block.

Next, kinetics related to DA release rates were examined using release velocity (rate of DA release per second between stimulation and peak; Fig. 5*D*) and the latency to reach peak [DA] (Fig. 5*E*). Release velocity showed clear differences between core controls and other groups as indicated by both a main effect of group [*F*_(3,425)_ = 67.01, *p* < 0.00001], and a group × block interaction [*F*_(9,425)_ = 3.13, *p* = 0.001]. Core controls showed significantly faster release velocity than each of the other groups at all blocks (all *p* < 0.00001), but no other groups differed from each other (all *p* > 0.10). Although all groups exhibited significant linear contrasts across blocks (all *p* < 0.0001), core controls showed more rapid increases in release velocity across blocks than core cocaine [*F*_(1,425)_ = 6.29, *p* = 0.01], Shell controls [*F*_(1,425)_ = 9.16, *p* = 0.003], and Shell cocaine [*F*_(1,425)_ = 17.09, *p* = 0.0004]. However, linear contrasts between shell controls and either cocaine group were not different (both *p* > 0.20).

Cocaine experience also reliably affected latency to reach peak [DA] (Fig. 5*E*). There was a main effect of group [*F*_(3,425)_ = 147.8, *p* < 0.0001] and a group × block interaction [*F*_(9,425)_ = 12.66, *p* < 0.0001]. Unlike the previous metrics, latency to peak showed the most profound changes in the shell rather than core after cocaine experience. Shell cocaine was significantly slower to reach peak than all other groups (all *p* < 0.00001), which was due to slowed rates in the low peak block compared with all other groups in that block (all *p* < 0.00001). However, the average response of the cocaine-experienced groups was remarkably similar to the shell; contrast between shell controls and the averaged cocaine groups was not significant [*F*_(1,425)_ = 1.31, *p* = 0.26], whereas a contrast comparing core controls to the cocaine groups was highly significant [*F*_(1,425)_ = 242.9, *p* < 0.00001]. Thus, for both releaser metrics, both cocaine groups were much closer to the shell controls in both rate and rates of change across blocks than core controls.

Finally, I examined how peak-grouped signals differed in reuptake dimensions including V_max_ (maximum rate of reuptake between peak and 20% decay from peak [T20]), FWHH (time between stimulation and 50% peak [DA] after peak), slope (change in DA between 20% decay from peak [T20] and 80% decay from peak [T80]), and latency to return to postpeak baseline (as determined by a 95% confidence interval around the prestimulation baseline).

V_max_ rates of reuptake mirrored those obtained from release velocity (Fig. 5*F*). Strong main effects of group [*F*_(3,425)_ = 43.11, *p* < 0.00001] and group × block interaction [*F*_(9,425)_ = 2.64, *p* = 0.006] were due almost exclusively to differences between core controls and all other groups (group-wise comparisons versus core control, all *p* < 0.00001). In contrast, there were no group-wise differences between shell controls and either of the cocaine groups (both *p* > 0.75). Likewise, the change in reuptake across blocks increased faster in core controls relative to each of the other groups (all linear contrast comparisons, *p* < 0.004), whereas these rates did not differ between shell controls and either of the cocaine groups (both *p* > 0.40).

In contrast, FWHH appeared to more closely resemble latency-to-peak measures (Fig. 5*G*). Again, there was a main effect of group [*F*_(3,425)_ = 116.1, *p* < 0.00001] and group × block interaction [*F*_(9,425)_ = 7.52, *p* < 0.00001], which was largely due to differences in slowed rates in the shell cocaine group compared with all other groups (all *p* < 0.00001). As with latency to peak, FWHH showed an interesting property in which the average cocaine response was reliably different from core controls using a linear contrast [*F*_(1,425)_ = 149.76, *p* < 0.00001], whereas the cocaine groups were not different from shell controls [*F*_(1,425)_ = 0.87, *p* = 0.39].

Reuptake during slope showed a significant main effect of group [*F*_(3,425)_ = 4.89, *p* = 0.003], but no interaction of group × block (*p* = 0.29; Fig. 5*H*). This modest effect appeared to be due to a significantly faster clearance rate in core controls than all the other groups (all pairwise comparisons vs. core control, *p* < 0.02), while there were no differences between either of the cocaine groups relative to the shell controls (*p* > 0.80).

Return to baseline latency was largely determined by region rather than drug experience (Fig. 5*I*). There was a main effect of group [*F*_(3,425)_ = 19.02, *p* < 0.0001], but no interaction of group × block (*p* = 0.06). This group effect was not due to drug condition within a region (core control vs. core cocaine, *p* = 0.08; shell control vs. shell cocaine, *p* = 0.10), but rather to slower baseline return in the shell than the core in both drug conditions (core control vs. shell control, *p* = 0.003; core cocaine vs. shell cocaine, *p* = 0.0001).

**Figure 6.**
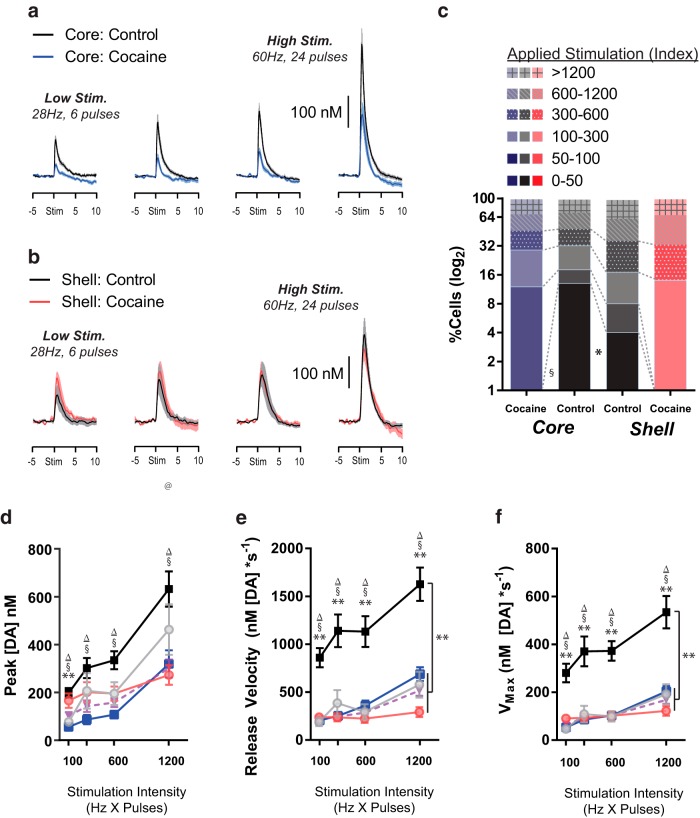
Average phasic DA release in the NAc core (***A***) and shell (***B***) of controls (black/gray) and cocaine self-administering rats (blue/red) in stimulation index–aligned bins. ***C***, For each drug group and region, the proportion of cells (of all observations) in each stimulation index bin. Note log_2_ scale used to show the loss specifically of the low stimulation index observations in the cocaine groups. Peak [DA] (***D***), rise velocity (***E***), and V_max_ (***F***) for treatment groups across stimulation intensity bins. **Control core vs. control shell; ^Δ^control core vs. both cocaines; ^§^control core vs. cocaine core; ^@^control shell vs. cocaine shell; ^‡^control shell vs. both cocaines; *p* < 0.01 (Bonferroni-corrected α for multiple comparisons).

For the final set of analyses, data were aligned by the intensity of the applied stimulation (stimulation index). In general, cocaine experience had distinctly different effects on how stimulations affected DA release across regions (for pairwise *t* test comparisons between groups, see Tables 3 and 4 for Bonferroni-corrected *p*-values). In the core (Fig. 6*A*), DA release was significantly decreased relative to controls with the same stimulation parameters, whereas in the shell (Fig. 6*B*), cocaine experience produced more subtle effects that impact the dynamic range of the DA response. Grouping data into blocks by stimulation index according to a scale that roughly doubled in intensity between blocks, there was an overall significant difference in distribution between groups, **χ** ^2^ = 48.25, *p* < 0.00001 (Fig. 6*C*). Follow-up tests indicated that core controls had more low-intensity stimulations than both core cocaine (stimulation index 0–50, **χ** ^2^ = 10.58, *p* = 0.001) and shell controls (stimulation index 0–50, **χ** ^2^ = 3.85, *p* < 0.05). In contrast, the core cocaine group showed similar numbers of observations at in the low-intensity range as shell controls (stimulation index 0–50, **χ** ^2^ = 3.02, not significant [n.s.]) and shell cocaine subjects (stimulation index 0–50, **χ** ^2^ = 0, n.s.). Likewise, there were no differences between shell control and shell cocaine subjects in this bin (stimulation index 0–50, **χ** ^2^ = 2.17, n.s.). At the high end of the stimulation intensity range, there were fewer stimulations in the core controls than the mean of the cocaine groups (stimulation index >600, **χ** ^2^ = 4.14, *p* = 0.04), while Shell controls showed similar numbers as the cocaine groups (stimulation index >600, **χ** ^2^ = 0.01, n.s.).

**Table 3.** Stimulation index–aligned pairwise comparisons (individual drug groups)

*p*-values (*t* test)	**Core (control) vs. Shell (control)**				**Core (control) vs. Core (cocaine)**				**Shell (control) vs. Shell (cocaine)**			
Stimulation index	**100**	**300**	**600**	**1200**	**100**	**300**	**600**	**1200**	**100**	**300**	**600**	**1200**
Peak [DA]	0.002*	0.96	*0.02*	0.48	<0.0001*	0.003*	0.0006*	0.009*	*0.02*	0.96	0.99	0.14
Frequency	0.97	0.002*	0.46	1.00	0.24	0.73	0.87	0.14	0.12	0.37	0.76	1.00
AUC	*0.01*	0.98	0.21	0.45	0.0006*	0.003*	0.002*	*0.02*	0.06	0.70	0.83	0.14
Rise velocity	0.0006*	0.006*	0.0003*	<0.0001*	0.0002*	0.0002*	0.006*	0.0006*	0.31	0.42	0.49	0.14
Latency peak	0.002*	<0.0001*	<0.0001*	<0.0001*	0.45	0.36	0.61	0.11	0.07	0.002*	0.005*	*0.01*
V_max_	0.002*	0.003*	<0.0001*	0.0003*	0.001*	0.001*	0.0002*	0.004*	0.01*	0.78	0.78	0.16
FWHH	<0.0001*	<0.0001*	<0.0001*	<0.0001*	0.15	0.52	0.59	0.09	0.89	0.05	0.003*	0.13
Slope (T20–T80)	0.001*	0.81	0.004*	0.26	0.009*	*0.03*	0.002*	0.09	0.004*	0.77	0.68	0.10
Baseline return	0.26	0.74	0.96	0.91	0.07	*0.01*	0.11	*0.03*	0.56	0.28	0.10	0.88
T20 latency	<0.0001*	<0.0001*	<0.0001*	<0.0001*	*0.02*	*0.32*	0.09	0.07	0.30	0.10	0.28	0.31
T80 latency	0.006*	0.25	*0.02*	0.0004*	0.53	0.06	0.56	0.09	0.21	0.72	0.66	0.18

Significance (*p*-value) of pairwise *t* tests at each stimulation index bin (low [100–300], medium-low [300–600], medium-high [600–1200], and high [>1200]) between core control and shell control (left), core control and core cocaine (middle), and shell control and shell cocaine (right). Bold italics: *p < 0.01 (significant after Bonferroni correction); italics only: *p* < 0.05 (not significant after Bonferroni correction).

**Table 4.** Stimulation index–aligned pairwise comparisons (collapsed drug groups)

*p*-values (*t* test)	**Core (control) vs. shell (control)**				**Core (control) vs. both cocaines**				**Shell (control) vs. both cocaines**			
Stimulation index	**100**	**300**	**600**	**1200**	**100**	**300**	**600**	**1200**	**100**	**300**	**600**	**1200**
Peak [DA]	0.002*	0.96	*0.02*	0.48	0.0008*	0.003*	0.0001*	0.0003*	0.36	0.37	0.41	0.07
Frequency	0.97	0.002*	0.46	1.00	0.06	0.36	0.90	0.26	0.21	0.54	0.55	0.36
AUC	*0.01*	0.98	0.21	0.45	*0.02*	*0.03*	0.007*	0.003*	0.51	*0.44*	0.31	*0.02*
Rise velocity	0.0006*	0.006*	0.0003*	<0.0001*	<0.0001*	<0.0001*	<0.0001*	<0.0001*	0.43	0.27	0.92	0.69
Latency peak	0.002*	<0.0001*	<0.0001*	<0.0001*	0.005*	0.002*	0.0005*	<0.0001*	0.92	0.52	0.33	0.10
V_max_	0.002*	0.003*	<0.0001*	0.0003*	0.0002*	<0.0001*	<0.0001*	<0.0001*	0.07	0.55	0.79	0.30
FWHH	<0.0001*	<0.0001*	<0.0001*	<0.0001*	0.02	0.001*	<0.0001*	<0.0001*	0.42	0.74	0.79	*0.02*
Slope (T20–T80)	0.001*	0.81	0.004*	0.26	0.002*	0.006*	<0.0001*	0.001*	0.007*	0.59	0.70	0.21
Baseline return	0.26	0.74	0.96	0.91	0.99	0.30	0.60	0.39	0.25	0.28	0.74	*0.03*
T20 latency	<0.0001*	<0.0001*	<0.0001*	<0.0001*	0.0004*	0.0007*	<0.0001*	<0.0001*	0.09	0.70	0.60	0.0003*
T80 latency	0.006*	0.25	*0.02*	0.0004*	0.83	0.51	*0.05*	0.23	*0.02*	0.10	0.75	0.07

Significance (*p*-value) of pairwise *t* tests at each stimulation index bin (low [100–300], medium-low [300–600], medium-high [600–1200], and high [>1200]) between core control and shell control (left; repeated from Table 3), core control and average of both cocaine groups (core and shell; middle), and shell control average of both cocaine groups (core and shell; right). Bold italics: *p < 0.01 (significant after Bonferroni correction); italics only: *p* < 0.05 (not significant after Bonferroni correction).

As above, several metrics were quantified to assess features of kinetics, although because the peaks were unequal, only a subset of measures was analyzed: peak [DA], release velocity, and V_max_. Consistent with peak-aligned measures above, cocaine experience shifted core DA release dynamics toward a more shell-like pattern across multiple metrics. For example, peak [DA] exhibited a main effect of group [*F*_(3,391)_ = 7.01, *p* = 0.0001] (Fig. 6*D*), which was due to significantly higher peaks overall in the core control group than both core cocaine (*p* = 0.001) and shell cocaine subjects (*p* = 0.02); Shell controls did not differ from either cocaine group (core cocaine, *p* = 0.09; shell cocaine, *p* = 0.41). Planned contrasts indicated that while both control groups exhibited significant linear increases in DA as a function of increasing stimulation index (core, *F*_(1,391)_ = 23.89, *p* < 0.00001; shell, *F*_(1,391)_ = 10.03, *p* = 0.002), core cocaine subjects showed a nearly significant trend in this direction [*F*_(1,391)_ = 3.74, *p* = 0.053], whereas shell cocaine subjects showed no relationship between stimulation and DA [*F*_(1,391)_ = 0.40, *p* = 0.53].

Similar patterns were found for release velocity (Fig. 6*E*) and V_max_ (Fig. 6*F*). Both showed significant main effects of group (release velocity *F*_(1,391)_ = 33.87, *p* < 0.00001; V_max_, *F*_(1,391)_ = 31.75, *p* < 0.00001), and both post hoc examinations revealed that core controls exhibited faster release and reuptake than each of the other groups (all *p* < 0.00001), whereas shell controls did not differ from either cocaine group (all *p* > 0.59). Indeed, planned contrasts indicated that only core controls displayed a linear correlation between applied stimulation and release [release velocity, *F*_(1,391_) = 14.91, *p* = 0.0001] and reuptake [V_max_, *F*_(1,391)_ = 14.43, *p* = 0.0002], while none of the other groups showed this correspondence (all *p* > 0.08).

## Discussion

Here, voltammetrically recorded rapid DA release was measured in the NAc core and shell after electrical stimulation of VTA afferents in freely moving rats. Although the present data replicate well-established differences between core and shell in normal animals (Jones et al., 1996; Mateo et al., 2005; Addy et al., 2010), abstinence from cocaine self-administration significantly altered this relationship. In general, cocaine-experienced subjects displayed DA release kinetics that became significantly more similar to normal shell kinetics regardless of region. Specifically, whereas core cocaine subjects displayed generally lower peak [DA], peak-matched stimulations produced slowed kinetic responses of both release and reuptake for cocaine rats relative to controls. In contrast, both shell cocaine and core cocaine subjects were often similar to shell controls on a wide variety of metrics regardless of whether the observations were aligned by peak or applied stimulation intensity. Collectively, these observations suggest that prior cocaine experience differentially alters DA terminal function in a region-specific manner, which likely has important ramifications for understanding altered neuroplasticity in cocaine-experienced populations, even long after the cessation of drug-taking behaviors.

To understand the function of normal phasic DA signaling in the brain, it is critical to consider a variety of factors including temporal dynamics of the signal, the neuroanatomical terminal region for DA afferents, and the behaviorally relevant task being encoded. There are well-known intrinsic differences in signaling kinetics between core and shell due to neuroanatomical features of these regions. For example, NAc shell expresses a decreased density in the DAT compared to the core, and as such, displays reliably slower synaptic reuptake of released DA (Jones et al., 1996). The present study replicates this previous work by demonstrating slower reuptake in the shell than core in controls by multiple metrics including V_max_, FWHH, slope, and the latency to return to baseline. These effects were largely true whether stimulations were aligned by stimulation parameters or peak DA response.

In addition to these reuptake measures, there were reliable differences in release kinetics between core and shell in controls, including faster release velocity and latency to peak. For example, frequency-aligned DA kinetics (e.g., release velocity, V_max_, peak [DA]) in the core linearly scaled with applied stimulations, whereas these same factors in the shell remained relatively flat regardless of stimulation intensity. This sensitivity of peak DA release arising from the intensity of impulse activity may support a functional role in normal behavioral task signaling. For example, in the NAc, core peak DA during predictive cues in a value-based decision-making task reliably scales with the animal’s preferred option when weighing cost–benefit choices, whereas DA release in the NAc shell showed similar DA peaks in the same conditions (Day et al., 2010; Sugam et al., 2012). Thus, a coupling between excitability and the magnitude of the DA response may indicate an intrinsic aspect of core DA signaling that encodes value by the relative peak for various stimuli (Saddoris et al., 2015b).

In contrast to these normal differences between core and shell, abstinence from cocaine self-administration induced a more homogeneous DA release pattern between subregions that were similar in several aspects to drug-naive shell kinetics and were consistent across multiple metrics and alignment properties. For the present study, both core cocaine and shell cocaine showed a peak-aligned distribution of responses that was statistically similar to shell controls, and which reliably differed from core controls. For example, although stimulations in core controls resulted in peak [DA] in the core that ranged between 40 and 1200 nm, stimulations in the shell controls and both cocaine groups produced peak DA release in the core that were primarily below 200 nm. Thus, cocaine experience appeared to shift the DA response in the core away from a widely dynamic response into a much narrower and smaller peak response typical of the shell, similar to recent findings obtained in a slice preparation (Siciliano et al., 2016).

While largely having more dramatic effects on core DA terminals, cocaine experience nonetheless induced some consistent changes in stimulated DA release in the shell as well. Here, DA release and reuptake kinetics (specifically, release velocity, latency to peak, and FWHH) were slower in cocaine rats than controls, but only at low levels of DA release (<200 nm). However, these lower peak DA responses are typical of the normal physiological range of peak [DA] observations (i.e., 40–150 nm) typically seen in freely moving rats in the NAc shell using an acute FSCV electrode (Aragona et al., 2008; Beyene et al., 2010; Wheeler et al., 2011; Cacciapaglia et al., 2012; Saddoris et al., 2015a). Thus, these somewhat limited effects may have significant ramifications for normal DA signaling during behavioral tasks. Further, stimulation-aligned data suggest that cocaine flattens the dynamic range of the DA response, with a generalized response at all applied stimulation intensities rather than a linear scaling of DA with stimulation changes.

Remarkably, the pattern of augmented DA release kinetics does not clearly mirror findings of dysfunctional DA signals during motivated learning behaviors (Spoelder et al., 2015; Saddoris et al., 2016b). In a recent finding, we showed that phasic DA release elicited by rewarding stimuli during associative learning was significantly impaired in both core and shell, though these deficits were distinct within subregion. In the core, peak DA in cocaine-experienced rats failed to differentially encode information about reward-predictive and irrelevant stimuli, instead displaying differences between cues several seconds after cue onset. Further, we found exaggerated DA release in the core during reward receipt in cocaine-experienced rats. In contrast, cocaine experience proved devastating to shell, where neither cues nor rewards elicited DA that was above baseline (Saddoris et al., 2016b).

Thus, while stimulated DA in the shell in the present study was less obviously affected by cocaine than in the core, phasic DA release in the shell during motivated behavior was profoundly impaired. This dissociation suggests that DA terminals in the shell remain functional, yet are unable to normally signal the significance of behavioral events. This inability to track behavioral stimuli despite relatively normal DA terminal function suggests a profound change in the mesolimbic circuitry induced by repeated cocaine experience, though whether this functional disconnection is due to changes in VTA inputs and/or local modulation of DA afferents has yet to be explored. In the core, however, there were some features during the learning task (Saddoris et al., 2016b) that complement the present finding. For example, DA signals in cocaine-experienced rats for the CS+ presentations was relatively sustained throughout the cue rather than briefly at cue onset, a dynamic more linked to the shell than core (Cacciapaglia et al., 2012; Saddoris et al., 2015a). Further, whereas DA signaling for predicted rewards by typically disappears in the core with training, consistent with reward prediction error hypotheses (Schultz et al., 1997; Pan et al., 2005), fully anticipated rewards persistently elicit large DA release events in cocaine-experienced rats (Saddoris et al., 2016b), a pattern of activity more typically found in the shell (Cacciapaglia et al., 2012; Saddoris et al., 2015a). Further, we have recently reported that shell (but not core) DA release in drug-naive rats tracks differences in reward magnitude, but in cocaine-experienced rats, this differential DA release pattern for reward magnitude is found in the core instead of the shell (Saddoris et al., 2016a). Thus, cocaine experience induces striking changes in the functional properties of the NAc core and shell which are differentially manifested in behavioral and synaptic properties in a region-specific fashion.

Collectively, these findings suggest that the core becomes more shell-like in its response dynamic to phasic DA signals after experience with cocaine self-administration. This hypothesis is consistent with previous reports showing that motivationally relevant encoding of relevant stimuli shifts dorsolaterally in the striatum in drug-experienced animals (Takahashi et al., 2007; Willuhn et al., 2012). These shifts are predicted by the anatomical organization of the mesolimbic system wherein complex “loops” of connections involving the striatum, limbic cortex, and midbrain result in learned information synapsing at increasingly dorsal and lateral targets within the circuitry over repeated experience (Haber et al., 2000, 2006; Haber, 2014). Indeed, disruption of earlier portions of these circuits can prevent these shifts in normal animals (Belin and Everitt, 2008; Belin et al., 2009; Willuhn et al., 2012), suggesting that dorsolateral shifts in encoding may reflect transitions to more habitual kinds of information (Robbins and Everitt, 2002).

Likewise, in cocaine-experienced rats, this dorsolateral shift appears to involve not just the neural output of the striatum, but also the DAergic input. This appearance of a functional dorsolateral shift in DA signaling properties may thus explain aspects of addiction as a chronically relapsing disorder; with functional changes in signaling along a dorsolateral axis within the striatum, representations of drugs and drug-associated stimuli may be encoded in a more habit-like manner and therefore more resilient against treatment. Indeed, we and others have shown that repeated drug intake biases animals toward a strong sign-tracking phenotype wherein outcome-associated stimuli take on abnormally high salience (McClory and Spear, 2014; Robinson et al., 2015; Spoelder et al., 2015; Saddoris et al., 2016b), and that sign-tracking responses are insensitive to changes in value of the associated outcome (i.e., more habit-like; Nasser et al., 2015).

In conclusion, the present findings provide evidence for a functional alteration in DA terminals for the core and shell in cocaine-experienced animals, patterns of which either reflect (core) or are distinct from (shell) behaviorally elicited DA signals. Future studies will investigate the causes for these neuroplastic changes and may provide insight into potential therapeutics to reverse these alterations.

## Synthesis

Reviewing Editor: Karen Szumlinski, University of California at Santa Barbara<br />Decisions are customarily a result of the Reviewing Editor and the peer reviewers coming together and discussing their recommendations until a consensus is reached. When revisions are invited, a fact-based synthesis statement explaining their decision and outlining what is needed to prepare a revision will be listed below. The following reviewer(s) agreed to reveal their identity: Erin Calipari. For some manuscripts transferred from JNeurosci, a decision is made to accept the manuscript without peer review and a brief statement to this effect will follow.

In consultation with two expert reviewers, we all agree that your report is suitable for publication in eNeuro. Both reviewers and I agree that the authors have addressed all of the major concerns put forth by the reviewers; however, we share the concern regarding the use of the term "shell-like". While appropriate for the discussion, this term should be removed from the title and de-emphasized. Upon this minor change, I will be happy to accept your article for publication.
